# Reconstitution of a eukaryotic replisome reveals suppression mechanisms that define leading/lagging strand operation

**DOI:** 10.7554/eLife.04988

**Published:** 2015-04-14

**Authors:** Roxana E Georgescu, Grant D Schauer, Nina Y Yao, Lance D Langston, Olga Yurieva, Dan Zhang, Jeff Finkelstein, Mike E O'Donnell

**Affiliations:** 1 DNA Replication Laboratory, Howard Hughes Medical Institute, Rockefeller University, New York, United States; Harvard Medical School, United States

**Keywords:** DNA replication, replication fork, CMG, Pol delta, Pol epsilon, *S. cerevisiae*

## Abstract

We have reconstituted a eukaryotic leading/lagging strand replisome comprising 31 distinct polypeptides. This study identifies a process unprecedented in bacterial replisomes. While bacteria and phage simply recruit polymerases to the fork, we find that suppression mechanisms are used to position the distinct eukaryotic polymerases on their respective strands. Hence, Pol ε is active with CMG on the leading strand, but it is unable to function on the lagging strand, even when Pol δ is not present. Conversely, Pol δ-PCNA is the only enzyme capable of extending Okazaki fragments in the presence of Pols ε and α. We have shown earlier that Pol δ-PCNA is suppressed on the leading strand with CMG (Georgescu et al., 2014). We propose that CMG, the 11-subunit helicase, is responsible for one or both of these suppression mechanisms that spatially control polymerase occupancy at the fork.

**DOI:** http://dx.doi.org/10.7554/eLife.04988.001

## eLife digest

Cells must replicate their DNA before they divide so that the newly formed cells can each receive a copy of the same genetic material. DNA replication requires complex molecular machinery called a replisome, which comprises multiple proteins, enzymes, and other molecules. First, an enzyme called a helicase starts to unwind the double-stranded DNA into two single strands. This process continues while other enzymes, called polymerases, use the exposed single strands as templates to make complementary new strands of DNA. One of these new strands is built continuously and called the ‘leading strand’. The other newly forming strand—the ‘lagging strand’—is made in the opposite direction, as a series of short fragments that are later joined together.

The replisomes in bacterial cells have been well studied, but many researchers are investigating the composition of the replisome in animals, plants, and fungi (collectively called eukaryotes). Now, Georgescu et al. have essentially rebuilt a eukaryotic replisome from 31 different proteins in a test tube and confirmed that it can make both leading and lagging DNA strands—just like in a normal cell. Further experiments revealed that the polymerase enzyme that operates on the leading strand cannot work on the lagging strand and vice versa. This exclusivity is unique to eukaryotic DNA replication, as bacterial polymerases can use either DNA strand as a template.

Georgescu et al. then found that the eukaryotic polymerases are actively prevented from copying the ‘wrong’ strand of DNA and suggest that the helicase enzyme that unwinds the DNA might be behind this activity. Important future studies must now address how the replisome deals with obstacles created by certain DNA-binding proteins and damaged DNA and how it interfaces with the molecules that control cell division and DNA repair.

**DOI:** http://dx.doi.org/10.7554/eLife.04988.002

## Introduction

Composition of the eukaryotic replisome and the function of its various proteins is an area of active investigation. Cellular studies reveal that eukaryotes use two different DNA polymerases for the leading and lagging strands, Pols ε and δ, respectively (Lee et al., 1989; Weinberg and Kelly, 1989; Tsurimoto et al., 1990; Waga and Stillman, 1998; Benkovic et al., 2001; Pursell et al., 2007; Kunkel and Burgers, 2008; Nick McElhinny et al., 2008; Stillman, 2008). Priming is performed by Pol α, a 4-subunit enzyme that contains an RNA primase and DNA polymerase activity and makes short RNA-DNA hybrid primers of 25–35 nucleotides (Kaguni and Lehman, 1988; Waga and Stillman, 1998; Benkovic et al., 2001; Stillman, 2008). The 11-subunit CMG complex consisting of the Mcm2-7 ‘motor’, a GINS heterotetramer, and one Cdc45 subunit (Moyer et al., 2006; Ilves et al., 2010; Costa et al., 2011; Costa et al., 2014) provides the helicase activity. Numerous other proteins travel with eukaryotic replication forks and have no bacterial homolog or known function. In addition, many replication fork-associated proteins undergo modifications in response to the cell cycle or DNA damage.

While in vitro synthesis of the leading strand replisome has been accomplished with the purified CMG complex from budding yeast (Georgescu et al., 2014), the discontinuous lagging strand is a much more difficult process and the number of proteins required for lagging strand synthesis is currently unknown. Indeed, epitope tagging of CMG subunits, followed by cell extract pull-outs and mass spectrometry, has identified a large ‘replisome progression complex’ (RPC) that contains CMG along with several other factors, some of which are essential for cell viability (Gambus et al., 2006; Gambus et al., 2009). Thus, RPCs contain CMG along with Mcm10, Ctf4, Pol α, Mrc1, Csm3, Tof1, FACT, and Topo I. Furthermore, there is evidence that nucleosomes may be involved in determining the size of eukaryotic lagging strand fragments (Smith and Whitehouse, 2012). We demonstrate here a 31 protein system, requiring the three replicative Pols α, ε, and δ, which performs both leading and lagging strand synthesis and generates Okazaki fragments of a size similar to those observed in cells.

Study of the eukaryotic replisome identifies a new process that has no precedent in bacterial systems. Bacteria use simple recruitment processes to attract and hold polymerases to the fork. These are typically mediated by polymerase interactions with other proteins at the replication fork, such as the helicase and sliding clamps (Benkovic et al., 2001). However, we find that in addition to recruitment processes that attract polymerases to the fork, eukaryotes use suppressive mechanisms, which prevent polymerase action on one strand or the other. Thus, while Pol ε extends the leading strand, its activity is suppressed on the lagging strand, even in the absence of Pol δ. In opposite fashion, we demonstrate here that Pol δ is active on the lagging strand in the presence of Pol ε and Pol α. This activity stands in contrast to the suppression of Pol δ on the leading strand shown in our earlier report (Georgescu et al., 2014). We also find that Pol α functions with CMG on both the leading and lagging strands. However, Pol α lacks a proofreading exonuclease and thus has lower fidelity than Pols ε and δ. Interestingly, Pol α extension activity is suppressed by Pol ε on the lagging strand, even though Pol ε is inactive on the lagging strand. Likewise, Pol δ suppresses Pol α on the leading strand despite its inefficient extension of this strand. Thus, multiple suppression reactions exist that prevent the activity of a polymerase positioned on the ‘wrong’ strand, and the only active solution is the asymmetric Pol ε/δ leading–lagging strand replisome with use of Pol α to prime the strands.

## Results

**Figure 1.**
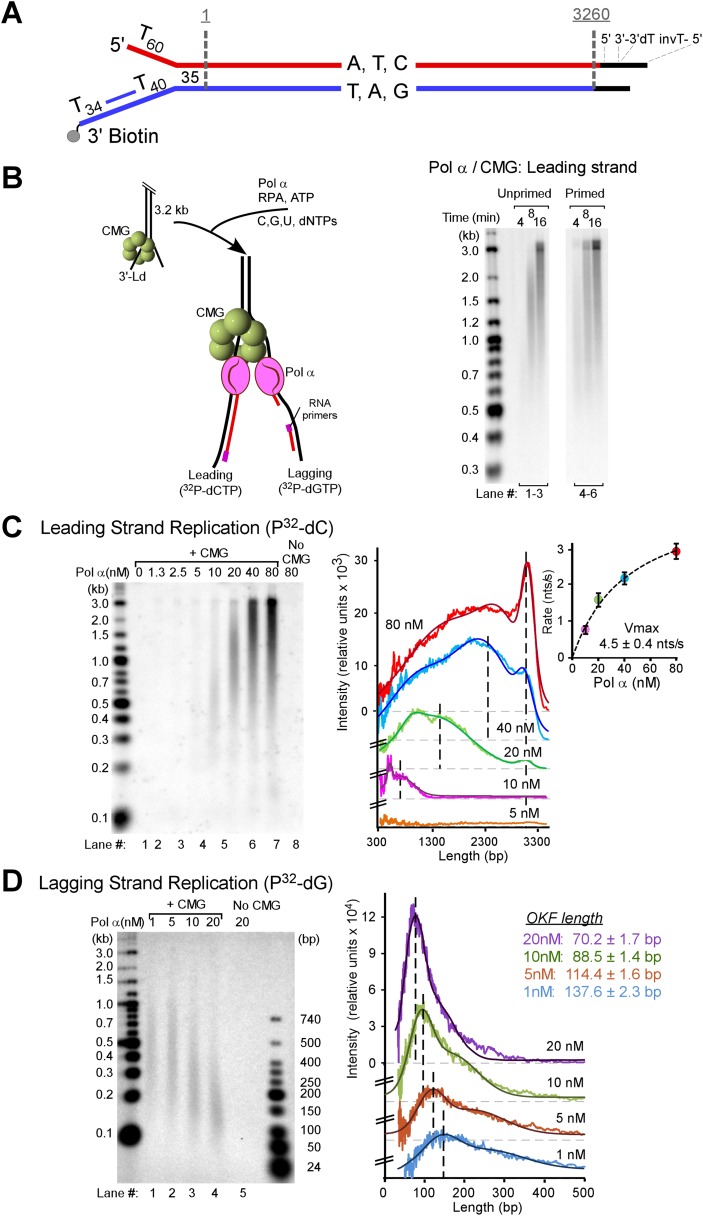
Pol α primes and extends leading and lagging strands with CMG helicase. (**A**) Scheme of the nucleotide-biased 3.2 kb fork substrate used in our experiments, explained further in ‘Materials and methods’. (**B**) *Left*: scheme of the assay. CMG is assembled onto the linear 3.2 kb forked DNA in the presence of 0.1 mM AMP-PNP, 60 μM dATP, followed by Pol α and RPA along with 5 mM ATP, 60 μM dTTP and 60 μM dGTP, 200 μM each rCTP, rUTP, rGTP, 20 μM dCTP, and 10 μCi ^32^P-dCTP, as described in ‘Materials and methods’. The nucleotide bias of the forked DNA enables labeling either the leading or lagging strand using ^32^P-dCTP or ^32^P-dGTP, respectively. *Right*: autoradiograph of leading strand products using unprimed (lanes 1–3) or primed (lanes 4–6) forked DNA. (**C**) *Left*: autoradiograph of leading strand replication products upon titrating Pol α into 20 min reactions. *Right*: scans of the gel lanes of leading strand products; the gel lanes were analyzed by Typhoon laser scanner, and the lane profiles were normalized to the corresponding molecular weight at each pixel in order to correct for the fact that longer products incorporate more radiolabel (α^32^P-dCTP) than shorter products. Replication reactions are plotted at the same scale. Each line trace was fit to a multiple Gaussian function, shown as a thin dashed line in each scan. The vertical dashed gray lines indicate the average rate of replisome progression at each concentration of Pol α examined. The inset graph plots the average replication rate vs the concentration of Pol α; the maximal rate (Vmax) of 4.5 ± 0.4 ntd/s was obtained by fitting of the data with a Michaelis–Menten-type equation. (**D**) *Left*: autoradiograph of lagging strand replication products using the indicated amounts of Pol α in 20 min reactions. *Right*: scans of the gel lanes of lagging strand Okazaki fragments showed in Figure 1D. Gel lanes were analyzed as described in (**C**). The average Okazaki fragment size obtained from the fit to the data is listed as an inset in the figure for each concentration of Pol α used. Omission reactions pertaining to Figure 1C,D are shown in Figure 1—figure supplement 1. **DOI:** http://dx.doi.org/10.7554/eLife.04988.003

We expressed and purified yeast CMG helicase in our previous studies and demonstrated its function in leading strand synthesis with Pol ε (Georgescu et al., 2014). The discontinuous lagging strand is a more difficult process than continuous leading strand synthesis, and the current study aims to identify how polymerases are coordinated during coupled leading–lagging strand synthesis. To facilitate this study, we designed a synthetic substrate of 3.2 kb that lacks dG on one strand and thus lacks dC on the other. We then ligated a synthetic fork onto one end (illustrated in Figure 1A). This nucleotide-biased fork enables separate monitoring of leading and lagging strand synthesis using either ^32^P-dCTP (leading) or ^32^P-dGTP (lagging).

**Figure 1—figure supplement 1.**
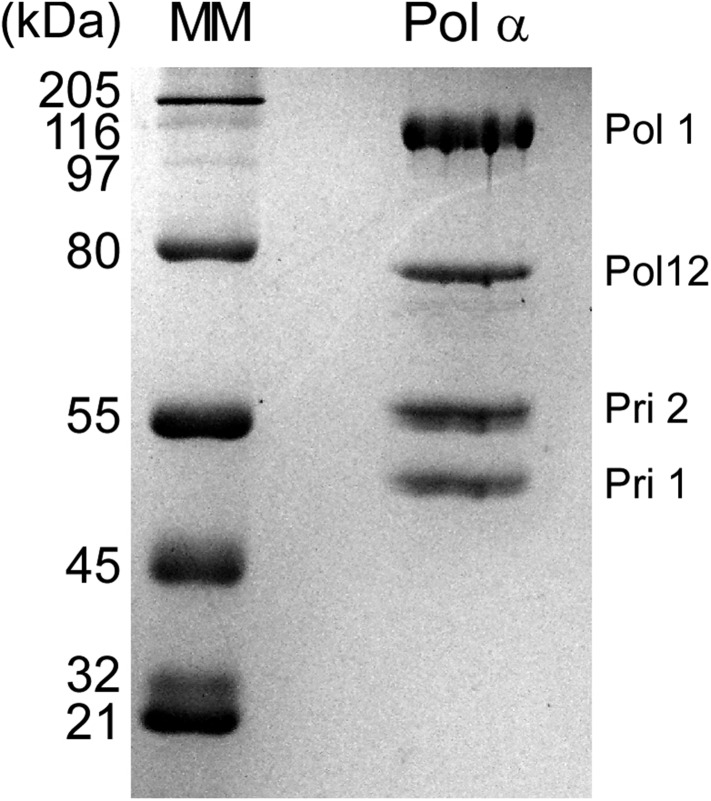
SDS-PAGE of purified Pol α. Pol α (2 μg) was analyzed in a 8% SDS polyacrylamide gel stained with Coomassie Brilliant Blue. The left lane, labeled ‘MM’ represents the Broad Molecular Weight markers (Bio-Rad). Subunits, labeled at the right, were confirmed by mass spectrometry analysis. The mass spectrometry analysis of the gel bands, and regions between bands, showed no trace of Ctf4 or Mcm10. **DOI:** http://dx.doi.org/10.7554/eLife.04988.004

**Figure 1—figure supplement 2.**
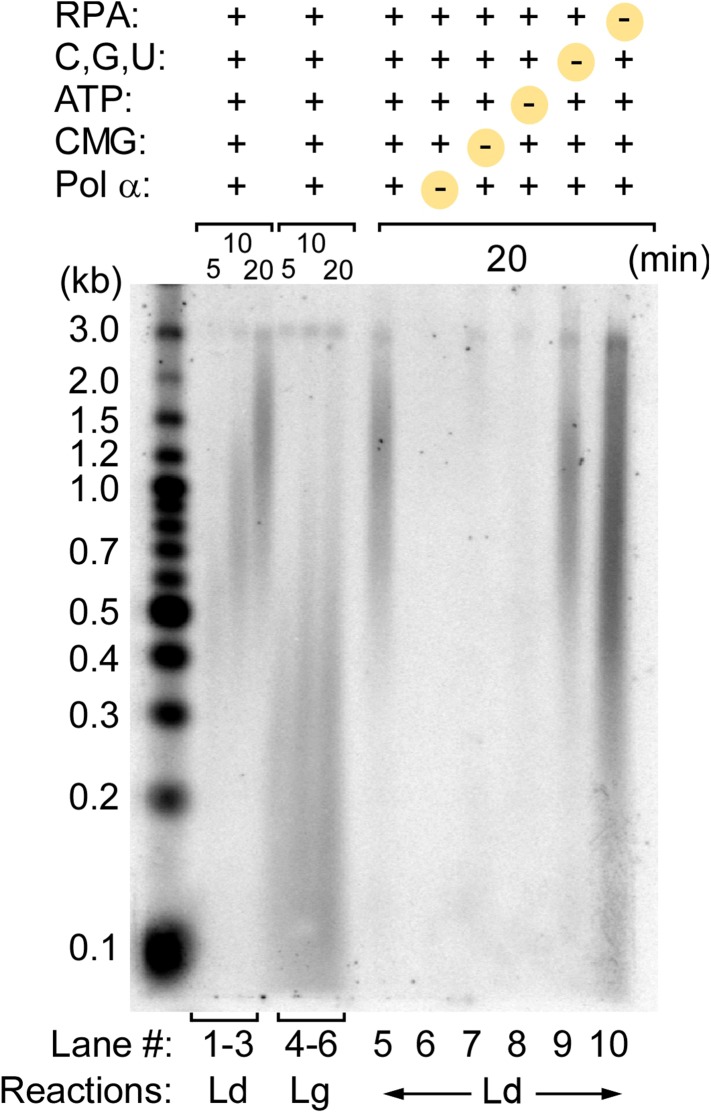
Pol α activity on the 3 kb forked DNA is dependent on CMG. Alkaline gel of omission assays for the Pol α, CMG, RPA leading strand replication reactions illustrated in Figure 1B,C. Lanes 1–6 show a time course of the complete replication reactions for the leading (lanes 1–3) and lagging strands (lanes 4–6). Lanes 5–10 illustrate leading strand products obtained upon omission of the indicated components at the top of the gel. **DOI:** http://dx.doi.org/10.7554/eLife.04988.005

### Pol α functions on both strands of a replication fork with CMG

Pol α is the eukaryotic primase and thus is required for lagging strand studies. To obtain the 4-subunit Pol α, we reconstituted it by expressing the Pol1 polymerase subunit of Pol α in yeast and Pol12 and the primase subunits, Pri1 and Pri2, in *Escherichia coli*. A mixture of cells containing the 4 subunits were lysed, and Pol α was purified as an intact 4-subunit holoenzyme (Figure 1—figure supplement 1). We then studied the behavior of Pol α with the 11-subunit CMG helicase and RPA on the forked DNA substrate. We initially assumed the lagging strand–specific Pol α would not function on the leading strand with CMG, especially since Pol δ-PCNA did not function well on the leading strand with CMG in our earlier study (Georgescu et al., 2014). To test Pol α function with CMG, we primed the leading strand with an oligonucleotide and examined Pol α DNA synthesis with CMG using ^32^P-dCTP (see scheme in Figure 1B). In contrast to our expectations, Pol α was highly active with CMG and completely extended the leading strand (Figure 1B, lanes 4–6). Dropout reactions demonstrated that CMG is absolutely required and therefore Pol α cannot perform this action alone (Figure 1—figure supplement 2).

**Figure 2.**
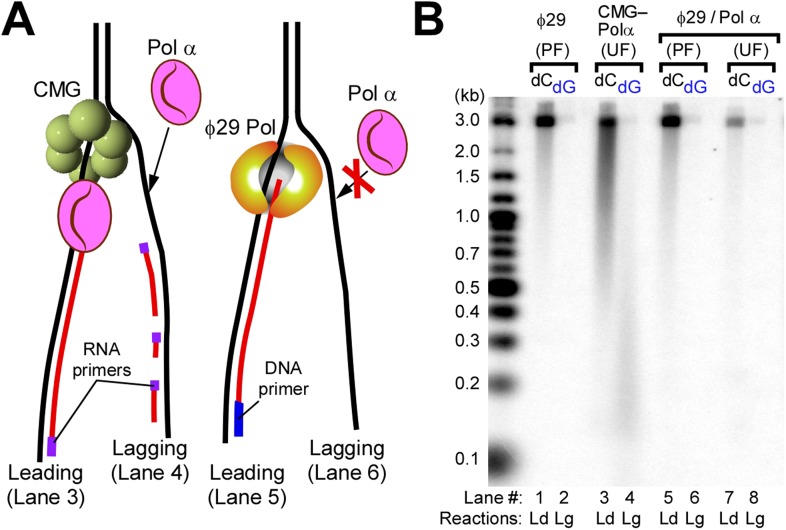
Pol α requires CMG for priming activity during unwinding of forked DNA. (**A**) Scheme of assays comparing Pol α activity using either CMG helicase or the strand displacing ϕ29 polymerase. (**B**) Autoradiograph of DNA products using either ^32^P-dCTP (leading) or ^32^P-dGTP (lagging). Use of a DNA-primed leading strand fork (PF) or an unprimed fork (UF) is indicated in the figure. Pol α was present at 10 nM, and reactions were for 20 min. Lanes 1 and 2 represent control reactions of ϕ29 polymerase alone. **DOI:** http://dx.doi.org/10.7554/eLife.04988.006

Models of priming at bidirectional origins in the SV40 and bacterial systems indicate that leading strand primers are formed on the lagging strand of one fork and then extended from the leading strand of the opposite fork (Waga and Stillman, 1998; Méndez and Stillman, 2003; Kaguni, 2011; O'Donnell et al., 2013). The current study uses a unidirectional linear fork and thus has no opposite fork from which to prime the leading strand. Therefore, we did not expect to observe leading strand synthesis using an unprimed leading strand fork. However, Pol α was fully active on unprimed DNA and thus the primase within Pol α is capable of priming the leading strand directly, while the polymerase subunit of Pol α is able to extend it (Figure 1B, lanes 1–3). We support and expand this observation again later in Figure 2.

A titration of Pol α into reactions with CMG shows an increasing rate of leading strand elongation as the Pol α concentration is raised, indicating that DNA synthesis by Pol α is distributive (autoradiograph illustrated in Figure 1C left panel and quantified on the right panel). The leading strand is continuously extended to full-length product; consequently, once Pol α has switched to the DNA elongation mode, it does not appear to switch back to the priming mode. This behavior has not previously been observed at a moving fork with CMG. Moreover, there are reports to suggest that the eukaryotic fork is discontinuous on both strands (reviewed in [Langston and O'Donnell, 2006]). The results of Figure 1, however, indicate that the leading strand of the eukaryotic replication fork is synthesized continuously under our assay conditions.

To determine whether this minimal replication system is competent to prime and extend the lagging strand, we utilized ^32^P-dGTP to specifically label lagging strand products. We assembled CMG on the forked DNA, then titrated Pol α into the reaction in the presence of RPA, dNTPs, and rNTPs. We expected to observe the small hybrid RNA/DNA 25–35 nucleotide primers known to be generated by Pol α (Kaguni and Lehman, 1988; Waga and Stillman, 1998; Benkovic et al., 2001; Stillman, 2008). However, we were surprised to observe sizeable Okazaki fragments (autoradiograph illustrated in Figure 1D left panel and quantified on the right panel). At 10–20 nM Pol α, a concentration consistent with intracellular concentrations of Pol α subunits (Ghaemmaghami et al., 2003; Kulak et al., 2014), Okazaki fragments were in the range of 200 bp, similar to their length in vivo (Smith and Whitehouse, 2012). The fact that the Okazaki fragment size decreases with increasing Pol α concentration indicates that priming is stochastic, occurring with lower frequency as the Pol α concentration is decreased (Figure 1D). These results demonstrate that small Okazaki fragments are intrinsic to Pol α action with CMG in the absence of nucleosomes.

### Pol α requires CMG for priming function

To determine if CMG is needed for Pol α priming function, we used ϕ29 DNA Pol, an efficient strand displacing enzyme (Blanco et al., 1989), to perform leading strand synthesis and generate a lagging single strand (ss) without CMG and asked whether Pol α primase could prime the lagging strand ssDNA template and produce Okazaki fragments with RPA present (illustrated in Figure 2A). The control shows ϕ29 Pol efficiently extends a primer through the 3.2 kb duplex (Figure 2B, lanes 1 and 5) and thus produces an ssDNA lagging strand. However, the lagging strand ssDNA did not serve as a template to generate Okazaki fragments when Pol α and RPA were present (Figure 2B, lane 6). In contrast, lagging strand synthesis by Pol α with CMG supports robust Okazaki fragment production by Pol α (lane 4). The requirement of CMG for Pol α primase activity suggests that Pol α forms a transient but specific interaction with CMG for priming action, since use of a heterologous enzyme to unwind DNA does not support Pol α priming activity. Although both Ctf4 and the essential Mcm10 proteins are known to bind Pol α (Gambus et al., 2009; Warren et al., 2009), these results demonstrate that neither Ctf4 nor Mcm10 are required for Pol α priming function with CMG in vitro. To support this observation, we examined our protein preparations for trace contaminating Ctf4 and/or Mcm10 by mass spectrometry, but no Ctf4 or Mcm10 was detected. Consistent with results of Figure 1B, Pol α also primes the leading strand directly and priming is stimulated by the presence of CMG (compare lanes 3 and 7). The results suggest Pol α binds CMG in the absence of Ctf4 or Mcm10 and therefore we tried to pull down a complex of Pol α with CMG but did not obtain a positive result. Thus the interaction, if it exists, may be weak, consistent with weak helicase–primase interactions in phage T4 and *E. coli* replication systems that can be deduced by activity assays but elude direct detection methods (Benkovic et al., 2001).

**Figure 3.**
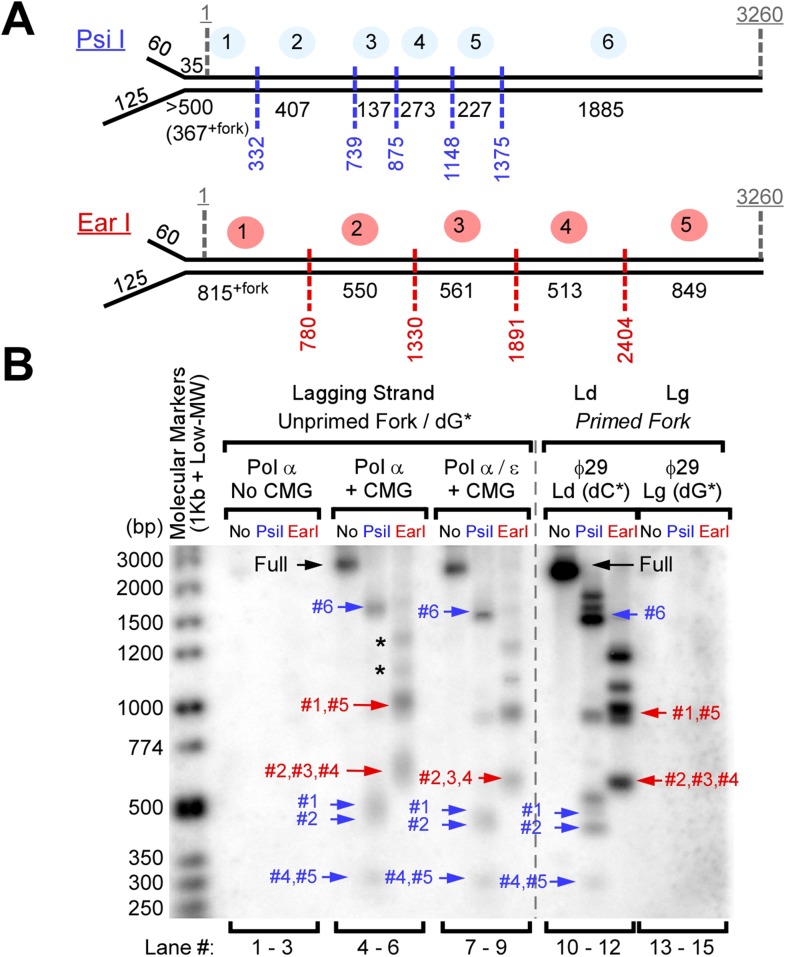
Okazaki Fragments are produced along the entire DNA. (**A**) Restriction enzyme map of the 3.2 kb substrate for Psi I and Ear I. (**B**) Lagging strand reactions were performed as detailed in ‘Materials and methods’ using an unprimed forked DNA, CMG, RPA, and either Pol α (lanes 4–6) or Pol α and Pol ε (lanes 7–9), then were either untreated (lanes 4, 7), treated with Psi I (lanes 5, 8), or treated with Ear I (lanes 6, 9). A control leading strand reaction using only ϕ29 Pol is shown in lanes 10–12. Pol α without CMG (lanes 1–3) and ϕ29 alone (lanes 13–15) gave no lagging strand products. The (*) mark incomplete digestion products. The reaction products were analyzed on a native 2% agarose gel. **DOI:** http://dx.doi.org/10.7554/eLife.04988.007

To determine if Okazaki fragments are distributed over the length of the DNA template, we used ^32^P-dGTP to label Okazaki fragments and then analyzed the distribution of radioactivity across the DNA by restriction enzyme analysis in a native gel (Figure 3). The analysis using CMG and Pol α shows that all the restriction fragments are radioactive and therefore Okazaki fragments are synthesized along the entire length of the DNA (lanes 4–9). To create size markers, ϕ29 Pol was used to extend the leading strand using ^32^P-dCTP, followed by restriction digestion (lanes 10–12). The analysis also confirms that Pol α cannot perform priming and extension in the absence of CMG (lanes 1–3) and that ϕ29 Pol cannot perform lagging strand synthesis (lanes 13–15).

### Pol ε switches with Pol α polymerase on the leading strand

**Figure 4.**
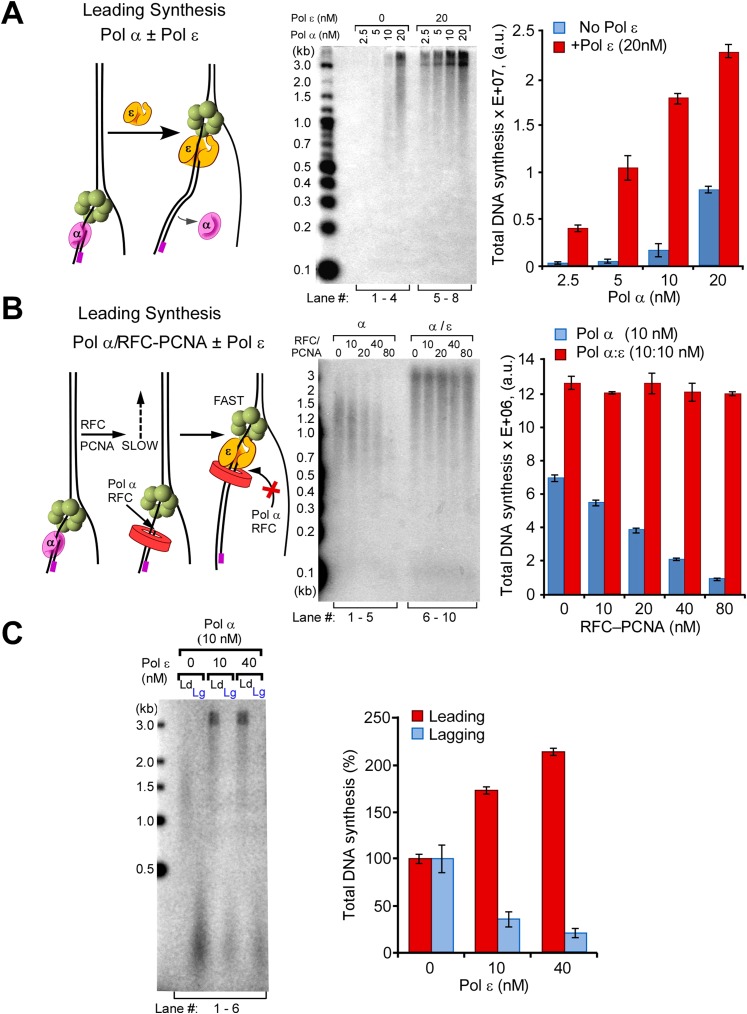
Pol ε switches with Pol α on the leading strand but is not active on the lagging strand. (**A**) *Left*: scheme of the assay. *Middle*: titration of Pol α into leading strand reactions in the absence of Pol ε (lanes 1–4) or in the presence of 20 nM Pol ε (lanes 5–8). The reactions were 20 min and contained unprimed DNA fork template. *Right*: histogram illustrating total DNA synthesis obtained from Typhoon laser scan analysis in Figure 4—figure supplement 1A (the error bars represent Standard Fit Errors obtained from the Gaussian fit analysis). (**B**) *Left*: scheme of the assay. Titration of RFC-PCNA into a primed leading strand assay containing 10 nM Pol α with or without 10 nM Pol ε. *Right*: RFC-PCNA inhibits Pol α (lanes 1–5) probably by competing for the 3′ terminus as illustrated. When present, Pol ε rapidly extends the leading strand and is not inhibited by RFC-PCNA (lanes 6–10). Replication reactions were performed in the presence of ^32^P-dCTP for 15 min. *Right*: histogram illustrating total DNA synthesis obtained from Typhoon laser scan analysis in Figure 4—figure supplement 1B (the error bars represent Standard Fit Errors obtained from the Gaussian fit analysis). (**C**) *Left*: leading and lagging strand synthesis is monitored in the same reaction plus or minus Pol ε. Each reaction was divided to separately monitor either the leading (^32^P-dCTP) or lagging (^32^P-dGTP) strand. Pol ε is absent in the reaction of lanes 1 and 2, and Pol ε is present in the reaction of lanes 3 and 4. *Right*: histogram illustrating total DNA synthesis obtained from Typhoon laser scan analysis in Figure 4—figure supplement 1C (the error bars represent Standard Fit Errors obtained from the Gaussian fit analysis). Lane analysis of the autoradiographs from panels A, B, and C are shown in Figure 4—figure supplement 1. **DOI:** http://dx.doi.org/10.7554/eLife.04988.008

Pol ε is the leading strand enzyme and presumably takes over the leading strand from Pol α after this distributive enzyme dissociates from DNA. To determine if the Pol α/ε switch occurs as expected, we preloaded CMG on the forked DNA, then added increasing amounts of Pol α either with or without Pol ε, and stopped the reactions after 20 min. If Pol ε takes over the leading strand from Pol α, full-length products will be observed sooner in reactions that contain Pol ε because in the presence of CMG, this enzyme synthesizes DNA faster than Pol α. The results show full-length product in all the lanes containing Pol ε with Pol α (autoradiograph in Figure 4A left, compare lanes 1–4 with 5–8; the quantification on the right panel is based on the autoradiograph analysis shown in Figure 4—figure supplement 1A). These observations indicate that after Pol α primes the DNA, Pol ε takes over and rapidly extends the leading strand. The reactions lack RFC/PCNA, but the presence of RFC/PCNA does not alter the outcome (Figure 4—figure supplement 2). At sufficiently high concentrations, RFC/PCNA competes with Pol α and suppress its extension activity with CMG, as previously reported without CMG (Mossi et al., 2000) (Figure 4B and quantification analysis shown in Figure 4—figure supplement 1B). The results show that RFC/PCNA also inhibits Pol α polymerase extension of primers on the lagging strand.

**Figure 4—figure supplement 1.**
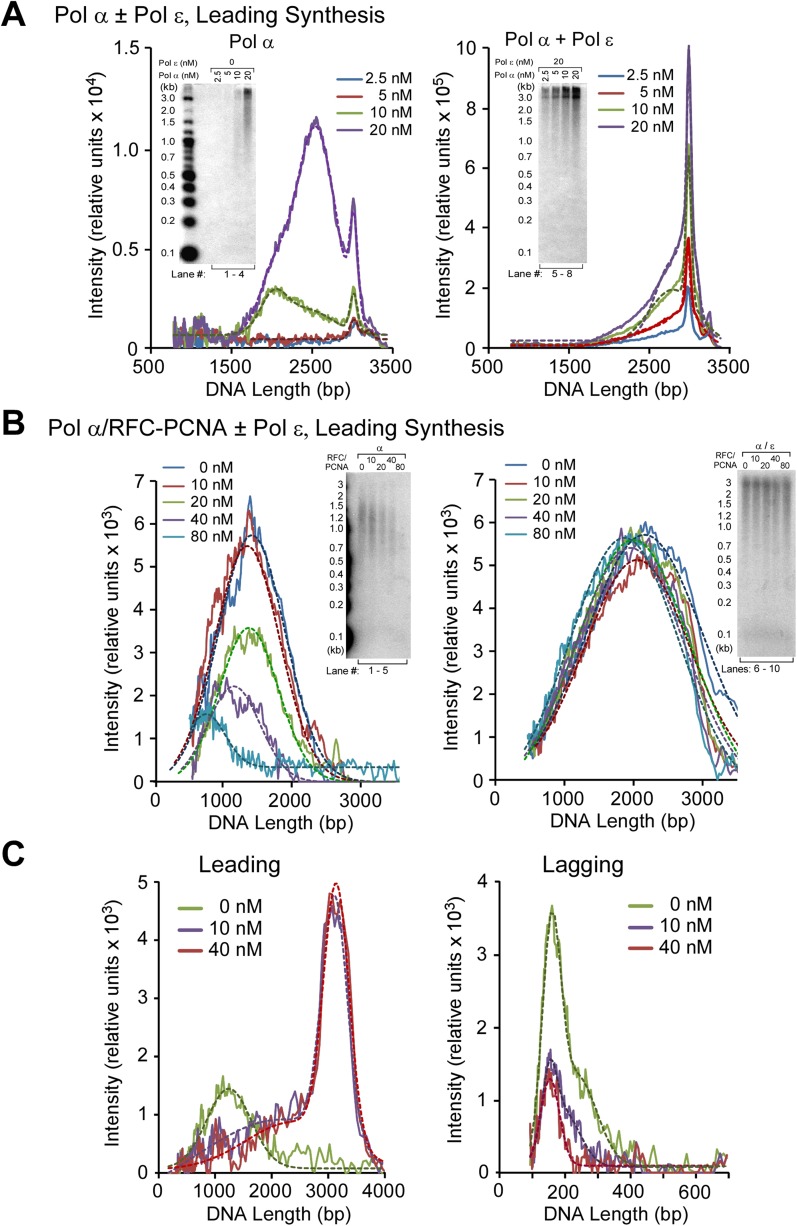
Analysis of replication products in Pol α and Pol α−ε titrations illustrated in Figure 4A–C. The autoradiographs shown in Figure 4 were analyzed by Typhoon laser scanner, and the lane profiles were normalized to the corresponding molecular weight at each pixel, as previously described (Kurth et al., 2013). This corrects for the fact that longer products incorporate more radiolabel (α^32^P-dCTP or α^32^P-dGTP) than shorter products. Each line trace was fit to a single or multiple Gaussian functions, shown as a thin dashed line in each scan. For ease of understanding, each of the panels **A**, **B**, and **C** correspond to the autoradiographs displayed in Figure 4A–4C, respectively (a cutout of the gels in Figure 4 are inserted, for ease of identification). **DOI:** http://dx.doi.org/10.7554/eLife.04988.009

**Figure 4—figure supplement 2.**
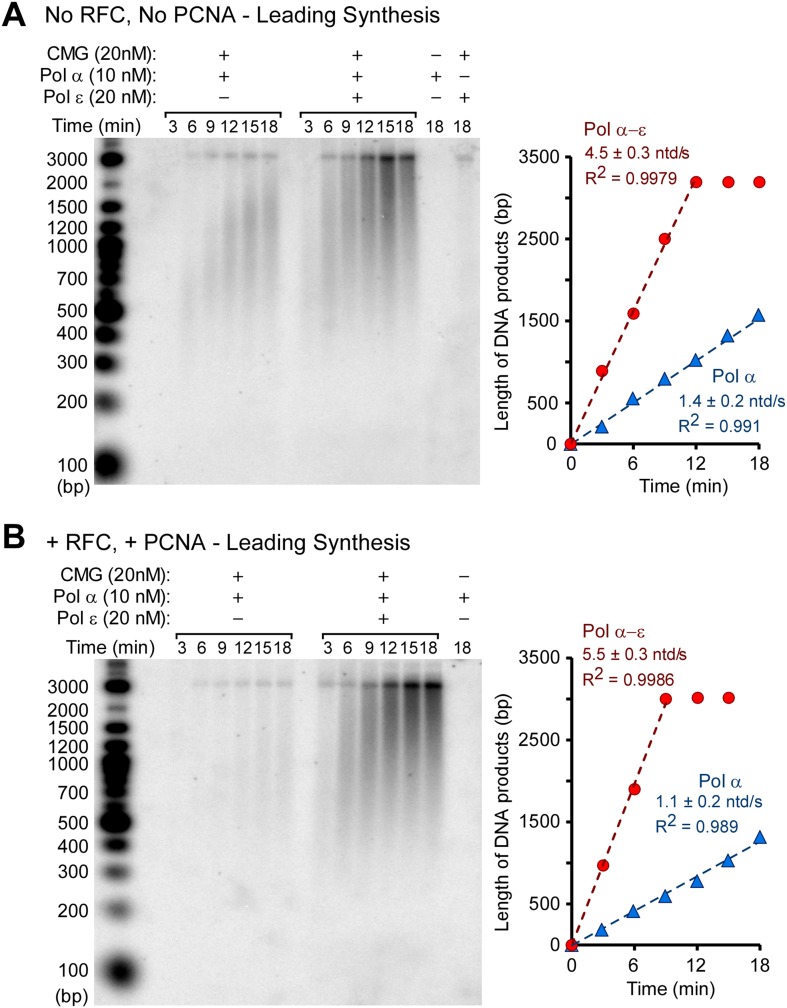
Pol ε excludes Pol α from the leading strand by taking over the primer whether RFC and PCNA are present or not. (**A**) Autoradiogram of CMG mediated Pol α extension of the leading strand on the unprimed 3.2 kb forked DNA, in the presence or absence of Pol ε, as indicated in the figure. Reactions were performed for the indicated times and otherwise performed as described in ‘Materials and methods’. The gel lanes were analyzed by Typhoon laser scanner (as in Figures 1, 4), and the plot on the right side quantitates the progression of DNA product length at the times indicated in the gel. The numbers in the plots represent the rate of the Pol α (blue) or Pol α-ε (red) obtained from the linear fit of the data points. (**B**) Autoradiogram of reactions performed as described in panel **A**, except 20 nM each of RFC and PCNA were present in all reactions. The plot on the right quantitates the data as described for panel **A**. **DOI:** http://dx.doi.org/10.7554/eLife.04988.010

**Figure 4—figure supplement 3.**
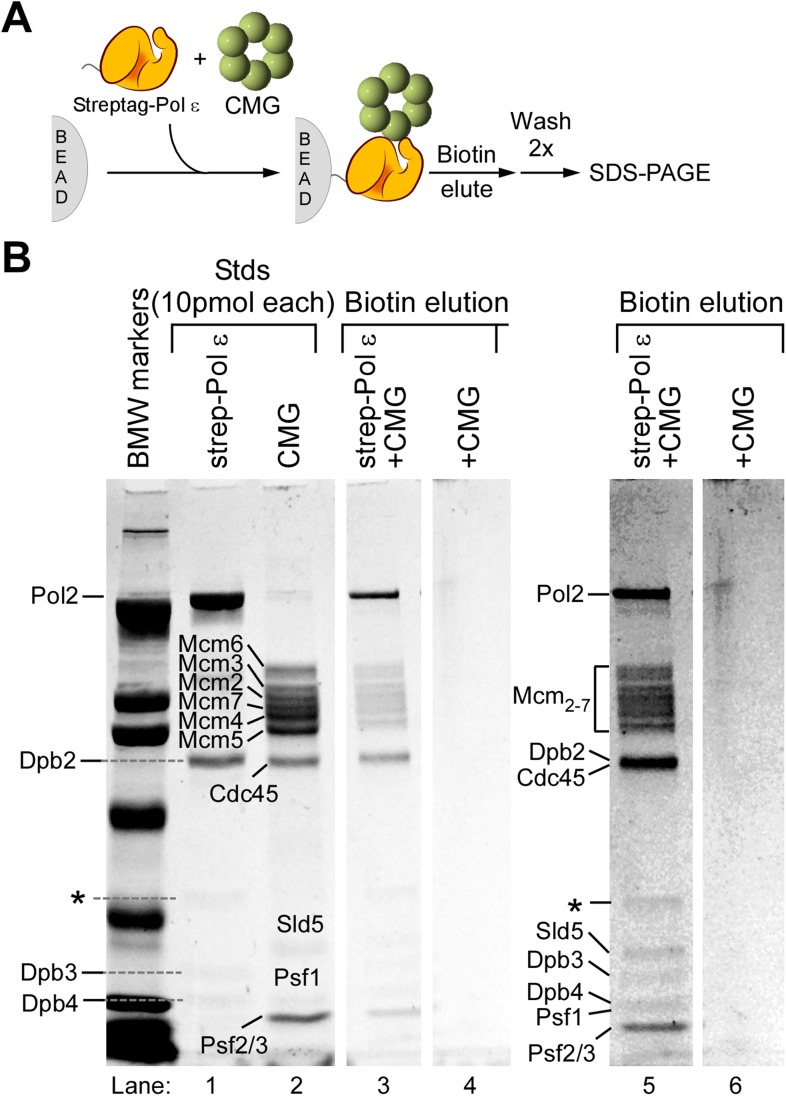
CMG and Pol ε form a stable CMGE complex. (**A**) Scheme of the bead assay to detect protein interactions. CMG-Pol ε complex was bound to Strep-Tactin magnetic beads via a strep-Pol ε, then washed and eluted with biotin; products were analyzed by 8% SDS-PAGE. Mixtures of proteins were as described in ‘Materials and methods’ and included 80 pmol strep-Pol ε and 120 pmol CMG. (**B**) SDS-Coomassie Blue stained PAGE of eluted protein complexes. The left lane, labeled ‘BMW markers’ represent the Broad Molecular Weight markers (Bio-Rad); lanes 1 and 2 show the protein preps used in the analysis. The location of each subunit is indicated. Cdc45 and Dpb2 co-migrate. The ‘*’ marks an impurity present in the strep-Pol ε prep. Lane 3 illustrates the biotin elution of the CMGE complex. Lane 4 represents the negative control in which the same amounts of CMG was mixed, in the absence of strep-Pol ε, then incubated with the beads, washed and eluted with biotin. The clear spaces between the last two lanes indicate these two lanes were taken from the same gel but were not adjacent to one another. Lanes 5 and 6 are a higher contrast of the lanes 3 and 4 to more clearly show the Sld5 and Psf1 subunits. **DOI:** http://dx.doi.org/10.7554/eLife.04988.011

The ability of Pol ε to suppress Pol α function with CMG might be facilitated by direct interaction of Pol ε with CMG, thus holding Pol ε at the leading strand primer terminus and preventing Pol α from binding. The Dpb2 subunit of Pol ε is known to bind the Psf1 subunit of GINS (Sengupta et al., 2013), and we have previously used a glycerol gradient to demonstrate that intact Pol ε can bind CMG helicase, forming a 1:1 CMG-Pol ε (CMGE) complex (Langston et al., 2014). In Figure 4—figure supplement 3, we document that the CMGE complex can also be reconstituted in a bead-based protein binding assay. In these experiments, we purified Pol ε containing an N-terminal StrepTag, incubated it with CMG, and then added Strep-Tactin magnetic beads. Biotin-specific elution of CMG in complex with StrepTag-Pol ε demonstrates that CMG is retained by Pol ε, forming the 15-protein ‘CMGE’ leading strand complex. No CMG was eluted from the column in the absence of Pol ε.

### Pol ε activity is suppressed specifically on the lagging strand

Considering that Pol ε takes over the primed template from Pol α on the leading strand, one may presume that Pol ε will also take over from Pol α on the lagging strand. In Figure 4C, we separately monitored leading and lagging strand synthesis in Pol α/CMG reactions in the absence or presence of Pol ε. The first two lanes are Pol α/CMG on the leading (lane 1) and lagging (lane 2) strands. At the concentration used, Pol α extended the leading strand DNA to about half of full length (lane 1), and the signal for Okazaki fragment synthesis was quite strong (lane 2). When Pol ε was added, it took over the leading strand and extended it full length (Figure 4C, lane 3); however, lagging strand Okazaki fragments were inhibited, a result we did not expect (lane 4). We also tested a higher concentration of Pol ε to see if more Pol ε was required to efficiently extend the Okazaki fragments, but lagging strand synthesis was not enhanced (Figure 4C, lane 6) (for quantification see Figure 4C right panel and Figure 4—figure supplement 1C). This result is contrary to conventional wisdom because one would normally expect more synthesis upon addition of more polymerase. This is especially true considering that Pol ε enhances the leading strand in the very same reaction in which it inhibits the lagging strand (i.e., the only difference is the radioisotope).

**Figure 5.**
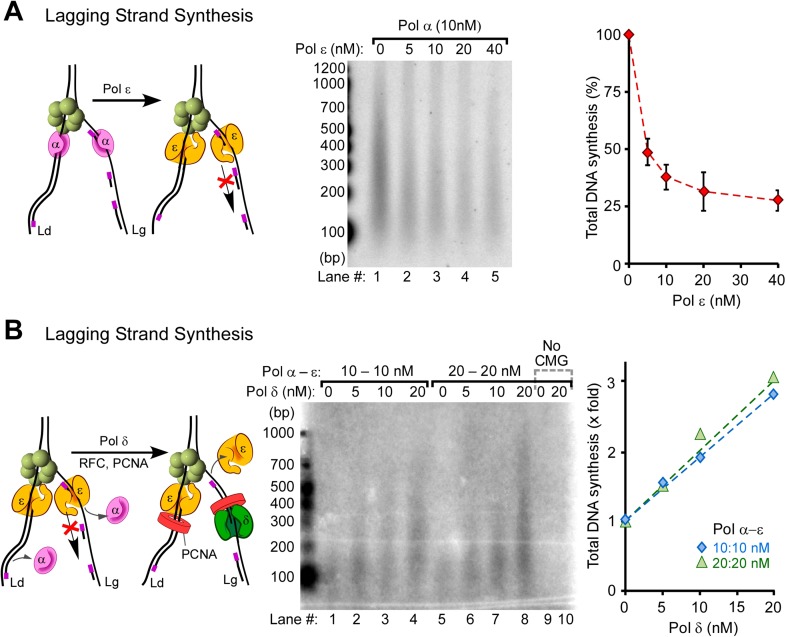
Pol δ functions on the ‘Pol ε suppressed’ lagging strand. (**A**) Titration of Pol ε into lagging strand reactions containing Pol α/CMG results in inhibition of the lagging strand in the absence of RFC-PCNA. Similar reactions containing RFC-PCNA give even more inhibition on the lagging strand (Figure 5—figure supplement 1). Reactions were for 20 min. (**B**) Pol δ is titrated into lagging strand reactions containing Pol α, Pol ε, RFC-PCNA, and CMG under conditions in which Pol ε and RFC-PCNA inhibit lagging strand synthesis. Lagging strand reactions (^32^P-dGTP) contain 10 nM each Pol α and Pol ε (lanes 1–4), or 20 nM each Pol α and Pol ε (lanes 5–8); RFC-PCNA are at 20 nM each. CMG concentration was 24 nM in all reactions. Lanes 9 and 10 are controls with no CMG but contain 20 nM each of Pol α, Pol ε, RFC, PCNA, and either no Pol δ (lane 9) or 20 nM Pol δ (lane 10). Reactions were for 20 min. The plots on the right of panels **A** and **B** represent quantifications of lagging strand replication reactions (using α−^32^P-dGTP) as described in the legend of Figure 4. **DOI:** http://dx.doi.org/10.7554/eLife.04988.012

The results of Figure 4C indicate that Pol ε activity is suppressed on the lagging strand. Moreover, Pol ε suppresses Pol α, suggesting that Pol ε gains access to lagging strand primed sites but is inactive on them. Considering that Pol ε extends DNA on RPA-coated primed ssDNA in the absence of CMG (Wold, 1997; Garg and Burgers, 2005; Georgescu et al., 2014), it is possible that CMG controls the activity of Pol ε on the lagging strand. We further supported this observation in Figure 5A by titrating Pol ε into Pol α priming reactions and monitoring lagging strand synthesis. As Pol ε is titrated into the reaction, Okazaki fragment synthesis is inhibited, confirming that Pol ε suppresses Pol α polymerase but is unable to extend lagging strand primers (see quantification in Figure 5A, right panel). Although Pol ε inhibits Pol α on the lagging strand, a combination of Pol ε and RFC/PCNA inhibits Pol α more than either Pol ε or RFC/PCNA alone (Figure 5—figure supplement 1A, lanes 1–2 and 4–7 vs lanes 8–11; quantification shown in Figure 5—figure supplement 1B). Inhibition of Pol α synthesis by RFC/PCNA is also consistent with results in the SV40 system (Tsurimoto and Stillman, 1991) and model studies using primed ssDNA (Figure 5—figure supplement 2) (Mossi et al., 2000). Hence, RFC/PCNA contributes to the observed inhibition of Pol α synthesis in addition to Pol ε.

**Figure 5—figure supplement 1.**
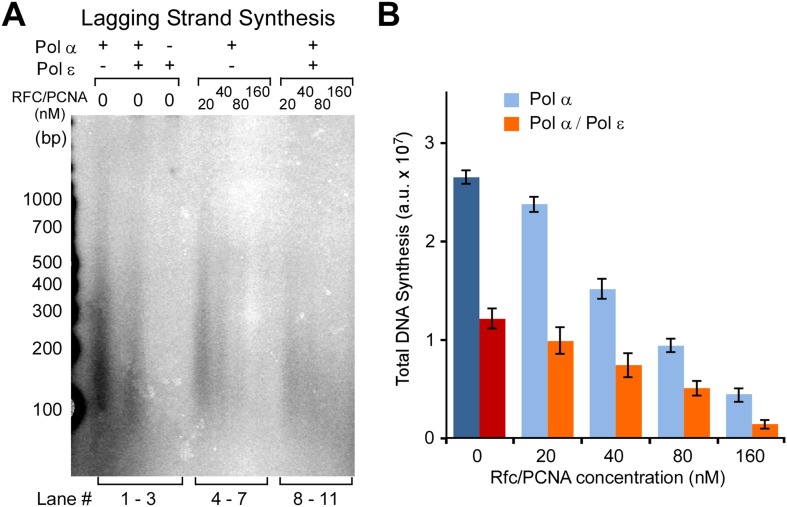
Pol ε and RFC/PCNA inhibit Pol α DNA polymerase on the lagging strand, but Pol ε cannot extend primed sites with or without RFC/PCNA. (**A**) Autoradiograph of lagging strand replication reactions using α^32^P-dGTP. CMG is pre-incubated and loaded on unprimed forked DNA, as described in ‘Materials and methods’, then either 10 nM Pol α (lane 1), 10 nM each Pol α and Pol ε (lane 2) or only 10 nM Pol ε (lane 3) are added along with RPA, dNTPs, and rNTPs; RFC-PCNA are absent. The Pol ε/CMG control shows no product synthesis on the lagging strand, as expected. The analysis of DNA products in lanes 1 and 2 show a 2.1 fold decrease in total DNA synthesis for the Pol α/Pol ε reaction relative to Pol α alone. Next, RFC-PCNA was titrated into reactions containing 10 nM Pol α with no Pol ε (lanes 4–7), or 10 nM each of Pol α and Pol ε (lanes 8–11). Reactions were 20 min. (**B**) Histogram illustrating total DNA synthesis obtained from Typhoon laser scan analysis of the autoradiograph in panel **A** (the error bars represent Standard Fit Errors obtained from the Gaussian fit analysis). **DOI:** http://dx.doi.org/10.7554/eLife.04988.013

**Figure 5—figure supplement 2.**
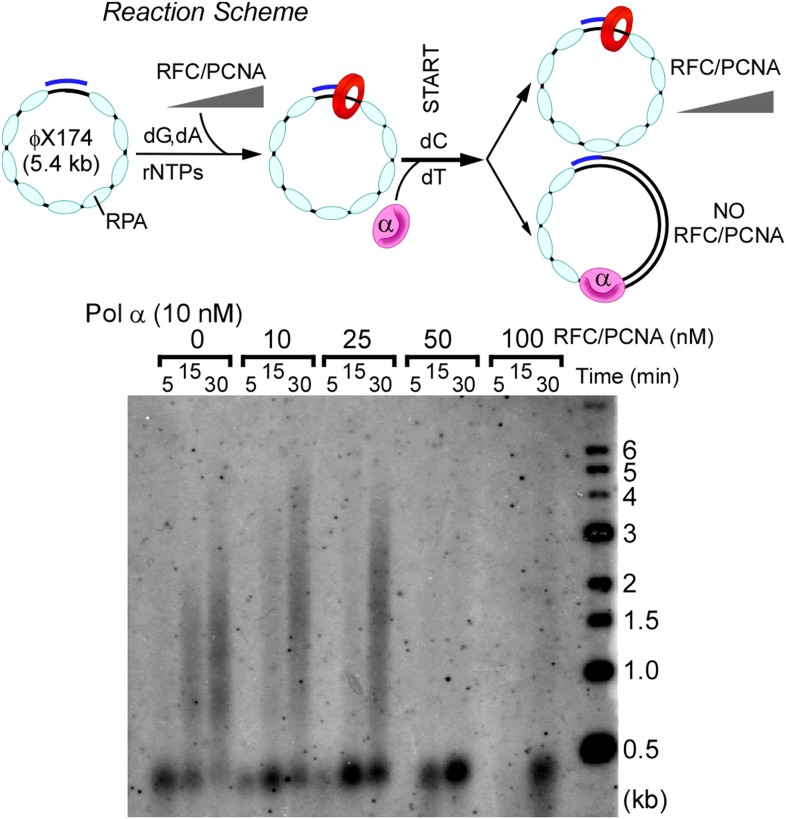
RFC and PCNA inhibit Pol α DNA polymerase activity on ssDNA model templates. Top: scheme of the assay. Primer extension assays utilized RPA-coated singly primed 5.4 kb φX174 ssDNA. RFC-PCNA is titrated into singly primed ssDNA replication assays containing 10 nM Pol α as described in ‘Materials and methods’. Concentrations of RFC, PCNA, and reaction times are indicated in the figure. **DOI:** http://dx.doi.org/10.7554/eLife.04988.014

### Pol δ-PCNA activity is suppressed on the leading strand but active on the lagging strand

**Figure 6.**
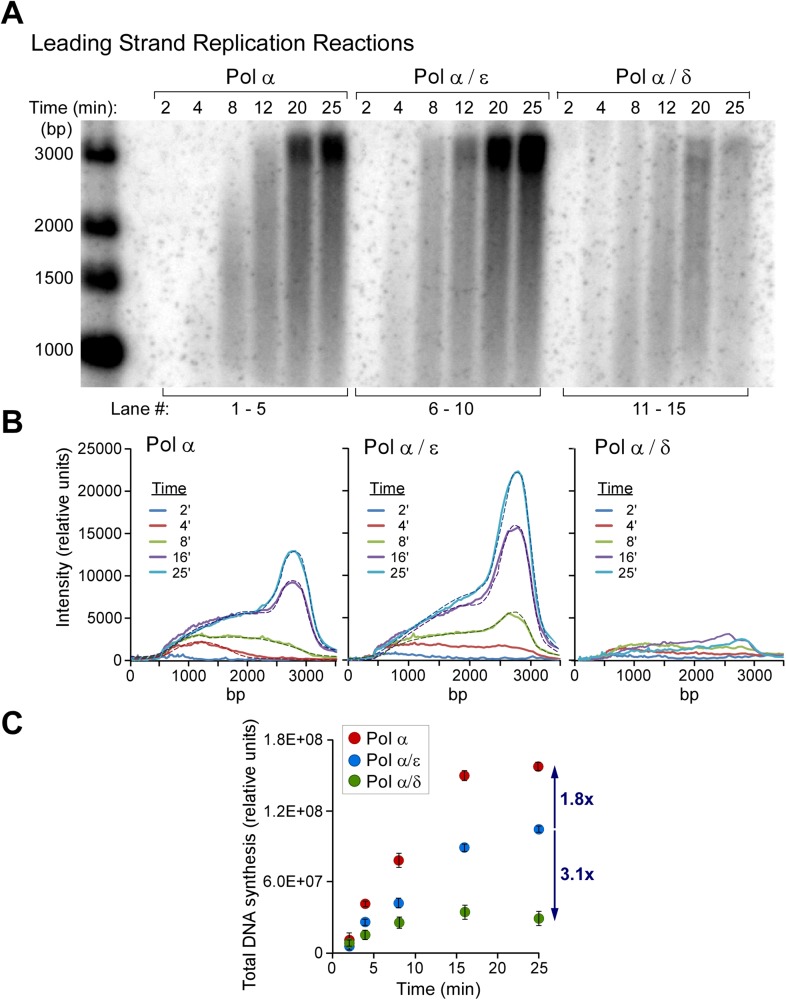
Polymerases switch with Pol α on the leading strand. (**A**) Alkaline agarose gel following the time course of leading strand extension using the indicated DNA polymerases. Reactions were assembled on unprimed forked DNA in presence of 24 nM CMG for 10 min before adding 15 nM Pol α (lanes 1–5), 15 nM each Pol α and Pol ε (lanes 6–10), and 15 nM each Pol α and Pol δ (lanes 11–15); all reactions contained 6 nM RFC and 20 nM PCNA. Reactions were initiated upon adding RPA and nucleotides as described in ‘Materials and methods’. The rates of Pol α reactions are high in this experiment because the amount of Pol α used here promotes relatively rapid fork progression as documented in Figure 1 and Figure 1—figure supplement 1. Still, the addition of Pol ε gives slightly faster forks due to the intrinsically faster rate of CMG-Pol ε over the rate of the distributive Pol α with CMG. (**B**) Autoradiograph quantification as described in the legend to Figure 4. (**C**) The analysis of DNA products at the end-point reaction (25 min) reveals a 1.8 fold increase in total DNA synthesis for the Pol α/Pol ε reaction relative to Pol α alone (lane 10 vs lane 5); the same comparison of total DNA synthesis in Pol α vs the Pol α/Pol δ reaction reveals a 3.2 fold decrease in total DNA synthesis (lane 15 vs 5). **DOI:** http://dx.doi.org/10.7554/eLife.04988.015

Our earlier study demonstrated that Pol δ-PCNA was slow and distributive on the leading strand in the presence of CMG and at 50 mM potassium glutamate (Georgescu et al., 2014). However, Pol δ-PCNA is highly processive during synthesis of the 5.4 kb primed RPA-coated ϕX174 ssDNA under the 50 mM potassium glutamate conditions used in the 3 kb replisome assays (Langston and O'Donnell, 2008). Pol δ-PCNA has been shown to be much less processive in reactions containing 125 mM added NaOAc (Chilkova et al., 2007). A high ionic strength is known to decrease processivity of enzymes, as the highly processive bacterial Pol III-β clamp replicase is decreased significantly at 100 mM NaCl (Griep and McHenry, 1989). Potassium glutamate is the physiological osmolyte in *E. coli* (Richey et al., 1987), but to the authors' knowledge, neither the intracellular ionic strength nor the major intracellular osmolytes are known for budding yeast. We do not know why Pol δ-PCNA is not very active on the leading strand with CMG. However, we have shown in this report that RFC/PCNA competes with Pol α on the leading strand (Figure 4B, lanes 1–5), indicating that PCNA is in fact loaded onto the leading strand. We demonstrate in Figure 6A that Pol δ added to the reaction exacerbates inhibition of Pol α in the presence of RFC/PCNA, while both Pol α and Pol ε are active in the presence of CMG and the amount of RFC/PCNA used (see quantifications in Figure 6B,C). Hence, Pol δ-PCNA does not function with CMG and severely competes and limits the function of Pol α with CMG. The addition of Pol δ to reactions containing Pols α and ε shows no significant effect on the leading strand reactions (Figure 6—figure supplement 1). The result of this three polymerase reaction is consistent with and anticipated from our earlier study of leading synthesis using primed forks (i.e., no Pol α) in which we demonstrate that Pol δ is inefficient and distributive on the leading strand and that Pol ε suppresses Pol δ by taking over the 3′ primed site in the CMG-dependent leading strand reaction (Georgescu et al., 2014).

**Figure 6—figure supplement 1.**
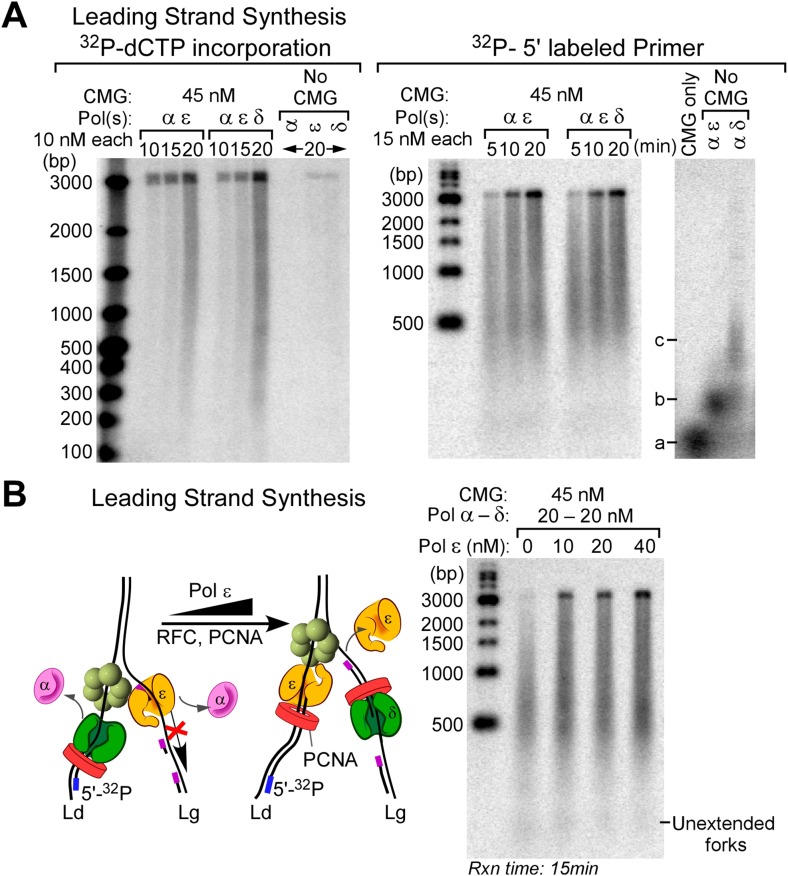
Pol δ does not inhibit the leading strand replication activity of Pol ε when all three polymerases are present. (**A**) Autoradiographs of leading strand replication reactions using α^32^P-dCTP (left) or ^32^P-5′ labeled primed fork (right). CMG is pre-incubated and loaded on DNA, as described in ‘Materials and methods’, then the different polymerases are added into the reaction at the specified concentrations along with RFC-PCNA, RPA, dNTPs, and rNTPs. There is a slight end-labeling product observed in the ^32^P-dCTP experiment (20′ control time points), and therefore, we repeated these reactions using the ^32^P-5′ labeled primed fork. At high CMG concentration and in the presence of all three polymerases, there is a very efficient replication of the forked DNA template. a, b and c mark the location of ^32^P-primers that are not extended, location of ^32^P-primers extended up to the fork junction, and location of a minority of ^32^P-primer extension by strand displacement activity of Pol δ in 20 min control reactions, respectively. (**B**) Pol ε functions on the ‘Pol δ suppressed’ leading strand. *Left:* scheme of the reaction. *Right:* Pol ε is titrated into leading strand reactions containing Pol α, Pol δ, RFC-PCNA, and CMG, and under these conditions, the Pol δ and RFC-PCNA inhibit leading strand synthesis by Pol α. The leading strand reactions contain 10 nM RFC, 20 nM PCNA, and the specified amounts of polymerases and CMG. **DOI:** http://dx.doi.org/10.7554/eLife.04988.016

Next, we tested whether Pol δ-PCNA could function on the lagging strand with CMG in the presence of Pol ε, Pol α, RFC, PCNA, and RPA, conditions in which Pol ε extends the leading strand with CMG but suppresses Okazaki fragment extension by Pol α. In Figure 5B, Pol δ was titrated into lagging strand reactions, and surprisingly, Pol δ was capable of extending Okazaki fragments under these circumstances. In fact, it is the only polymerase that we observed to perform this function. Hence, CMG does not prevent Pol δ function on the lagging strand, even while Pol δ-PCNA activity is suppressed on the leading strand.

### Leading and lagging strand synthesis occurs at similar rates

In order to compare total leading and lagging strand synthesis rates, we setup standard replication reactions by loading CMG onto the nucleotide-biased forked DNA, followed by the addition of all three DNA polymerases in the presence of RFC and PCNA; the reactions were divided, and replication was initiated by the addition of either ^32^P-dCTP or ^32^P-dGTP along with ATP, RPA, dNTPs, and rNTPS (see ‘Materials and methods’ for experimental details).

**Figure 7.**
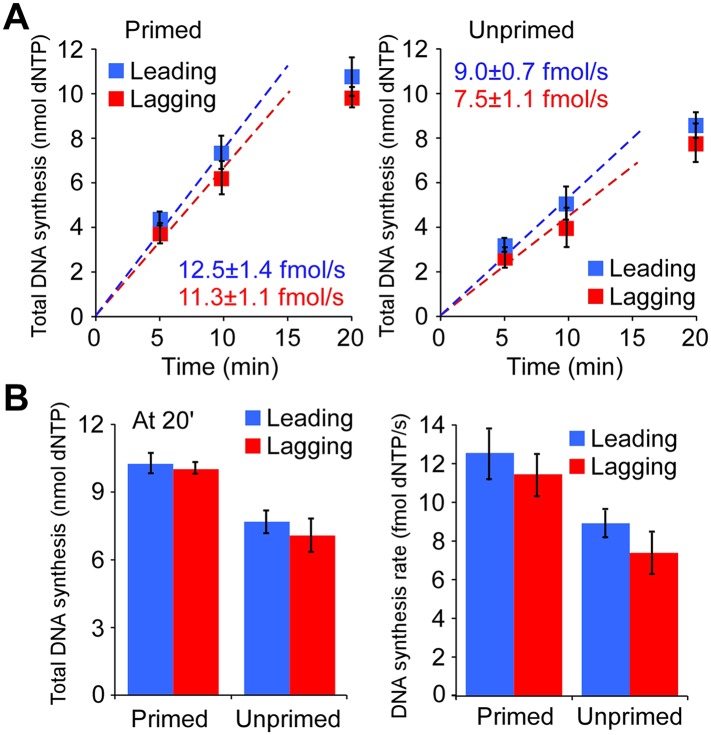
The leading and lagging strands are replicated at similar rates. (**A**) Time course of leading–lagging strand replication reactions with all three polymerases at 10 nM each using either a pre-primed fork (Left panel) or unprimed fork (Right panel). Experiments were performed in triplicate, using either ^32^Pα-dCTP or ^32^Pα-dGTP for leading–lagging replication reactions, respectively (for experimental details, see ‘Materials and methods’ section). The numbers shown represent the rate of incorporation (fmol dNTPs/s) and the SE obtained from the linear fit. (**B**) *Left*: comparative histogram depicting total DNA synthesis at the 20 min time point; *Right*: comparative histogram of the rate of incorporation of leading–lagging strand replication reactions on pre-primed and unprimed forked DNA substrates. **DOI:** http://dx.doi.org/10.7554/eLife.04988.017

Quantification of leading and lagging strand synthesis using either ^32^P-dCTP (leading) or ^32^P-dGTP (lagging) in the three polymerase replisome system shows similar amounts of leading and lagging strand synthesis (Figure 7). We tested the rate of dNTP incorporation using primed as well as unprimed forked DNA templates. While leading and lagging strand rates are similar using either DNA forked substrate, there is 20–25% more synthesis using primed DNA forks relative to unprimed DNA forks. We presume this difference reflects the additional time required for Pol α to prime the leading strand on the unprimed fork DNA, relative to use of forks that are pre-primed with a DNA oligonucleotide (i.e., primed forks).

## Discussion

The current report is the first reconstitution of a eukaryotic tri-polymerase leading/lagging strand replisome from purified proteins. Our earlier study used CMG with Pol ε to reconstitute leading strand replication (Georgescu et al., 2014). The continuous nature of the leading strand is relatively simple compared to the discontinuous process of the lagging strand. Indeed, PCNA and RFC were not even required for the leading strand (Georgescu et al., 2014). There are a multitude of proteins that travel with eukaryotic replication forks, many of which have been identified in the large RPC assembly (Gambus et al., 2006, 2009). This report demonstrates that leading/lagging strand replication is recapitulated in vitro with CMG, Pol ε, Pol α, Pol δ, RFC, PCNA, and RPA, comprising 31 different proteins. Hence, nucleosomes are not needed to size Okazaki fragments in vitro, the essential Mcm10 protein that binds Pol α is not required, nor is the Ctf4 trimer that connects Pol α to CMG (Simon et al., 2014). Study of the leading/lagging strand reaction revealed many facets of replication that were unanticipated, as summarized below.

### Suppression reactions, not recruitment specifies eukaryotic replisome architecture

The current report reveals that suppression reactions specify correct polymerase placement at the fork and that when a polymerase occupies the ‘wrong strand’ it is excluded from functioning. This stands in contrast to current views in which the mechanism of polymerase placement is thought to be via recruitment (i.e., each polymerase binds a particular protein on each strand). Indeed, polymerase recruitment by binding clamps and the helicase underlies attraction of polymerases to replication forks of bacteria, its phages, and the SV40 virus. Recruitment is also partly responsible for polymerase placement in eukaryotes. For example, Pol ε binds directly to CMG helicase, stabilizing it on the leading strand (Langston et al., 2014). However, the current study reveals the importance of suppression of polymerase action to the specific use of Pol ε and Pol δ on the leading and lagging strands, respectively. Thus, Pol ε function is suppressed on the lagging strand, even when Pol δ is not present. Likewise, our earlier study, confirmed here, showed that Pol δ-PCNA function is suppressed on the leading strand, even in the absence of Pol ε (Georgescu et al., 2014).

We were surprised to find that Pol α polymerase activity is highly functional with CMG on both leading and lagging strands in the absence of other polymerases. Pol α lacks the high fidelity of Pols ε and δ and does not provide bulk leading or lagging strand synthesis in cells, and thus processes must exist that suppress the polymerase activity of Pol α. In fact, we find many ways that Pol α polymerase is suppressed. One mechanism is by Pol ε positioning on the lagging strand. Interestingly, Pol ε is also suppressed on the lagging strand; perhaps, Pol ε is suppressed from function on the lagging strand by the relative orientation in which CMG holds Pol ε. On the leading strand, Pol ε simply prevents Pol α polymerase extension by switching with it and becoming processive with CMG. This can be likened to the switch of Pol δ with Pol α that prevents leading strand synthesis by Pol α in the SV40 system (Tsurimoto and Stillman, 1991). Pol α polymerase activity is also inhibited by RFC/PCNA as shown in this report and an earlier study (Mossi et al., 2000).

**Figure 8.**
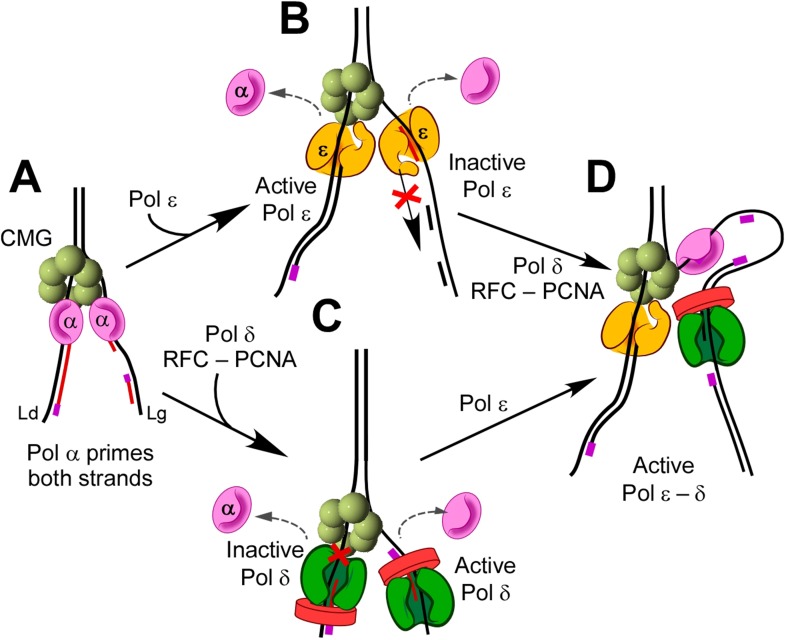
Exclusion reactions specify polymerase action at the eukaryotic fork. (**A**) Pol α interacts with CMG to prime the leading and lagging strands. Pol α can extend DNA on both strands with CMG in vitro, but it lacks high fidelity and does not replicate bulk DNA in vivo. (**B**) Pol ε can switch with Pol α on both strands, in the presence or absence of RFC/PCNA, but Pol ε is not active on the lagging strand. (**C**) Pol δ/RFC/PCNA can switch with Pol α on both strands, but Pol δ is inactive with CMG on the leading strand. (**D**) Presence of all three polymerases, Pols α, δ, and ε, provides active leading/lagging strand synthesis. **DOI:** http://dx.doi.org/10.7554/eLife.04988.018

The exclusion processes that underlie eukaryotic fork leading/lagging strand function are summarized in Figure 8. Pol α primes both leading and lagging strands (diagram A). Pol ε prevents further polymerase activity of Pol α by trading places with it, probably by waiting for the distributive Pol α to dissociate from DNA (diagram B); Pol ε then takes over the leading strand. Diagram C illustrates that Pol δ/RFC/PCNA also switch with Pol α but that Pol δ-PCNA cannot function in the presence of CMG on the leading strand. However, Pol δ-PCNA is uniquely capable of extending Okazaki fragments in the complete system (diagram D). Hence, the three-polymerase-CMG replisome is the product of polymerase suppression reactions that enable a unique asymmetric arrangement of DNA polymerases to advance the replication fork.

### Possible mechanisms of the polymerase suppression reactions

Yeast Pol δ is highly processive with PCNA on primed ssDNA under the conditions of this report (Langston and O'Donnell, 2008), yet Pol δ-PCNA is not effective in extending the leading strand with CMG helicase. The distributive behavior of Pol δ-PCNA with CMG suggests that CMG exerts a negative effect upon Pol δ, causing it to frequently dissociate from PCNA/DNA. A possible explanation for how CMG may cause distributive behavior of Pol δ with PCNA/DNA lies in our earlier finding that Pol δ self-ejects from PCNA upon completing replication of a template (Langston and O'Donnell, 2008). This property was originally noted for the highly processive *E. coli* Pol III replicase (O'Donnell, 1987; Studwell et al., 1989). Pol III remains firmly attached to the β clamp until reaching the last nucleotide (Stukenberg et al., 1994). This also holds true for T4 polymerase upon colliding with a hairpin (Hacker and Alberts, 1994). Self-ejection of polymerase is thought to be useful for recycling among numerous lagging strand primers (Benkovic et al., 2001). In eukaryotes, however, the self-ejection process may be used to promote polymerase asymmetry at the fork. A straightforward mechanism could be that CMG occludes the leading strand ssDNA, tricking Pol δ-PCNA to eject as if it were at the end of an Okazaki fragment. If CMG were to trigger the Pol δ−PCNA self-ejection process, it would result in distributive behavior of Pol δ-PCNA, suppressing its activity on the leading strand. Another possible mechanism could be that the 3′ terminus of the leading strand is sequestered and held in a posture that Pol δ cannot freely access.

The suppression of Pol ε on the lagging strand could possibly result from the geometry of CMG-Pol ε (CMGE) complex, directing the single Pol ε molecule to the leading strand. Suppression of Pol α by Pol ε on the lagging strand could perhaps be explained by competition of these polymerases for CMG, where Pol ε is suppressed from extending the primer. In this connection, the Pol2 gene encoding the catalytic polymerase actually contains two polymerase structures: the active polymerase/exonuclease on the N-terminal half and B-family polymerase in the C-terminal half of Pol2 that is presumed to be inactive (Tahirov et al., 2009). One possible mechanism by which Pol ε may suppress synthesis on the lagging strand could be that CMG positions Pol ε such that the ‘inactive polymerase’ binds lagging strand primers, preventing their elongation by Pol α and Pol ε. Interestingly, genetic studies have shown that the C-terminal ‘inactive polymerase’ region of Pol ε is required for cell viability, while the N-terminal region containing the active polymerase is dispensable (Dua et al., 1999; Kesti et al., 1999). The binding of Pol ε to CMG involves Dpb2, and possibly the Dpb3, Dpb4 subunits of Pol ε, and regions of Pol2 as well. Expression and purification of a mutant Pol ε that lacks the active N-terminal half of Pol2 (ΔN-Pol ε) yields a 4-subunit complex, while the active polymerase half of Pol ε is monomeric (Hogg et al., 2014). Hence, the ΔN-Pol ε probably connects to CMG similar to wild-type Pol ε and may carry out a vital function at the fork. Alternatively, the C-terminal portion of Pol2 could be important to an origin activation step prior to replisome formation. Although direct evidence is lacking, Pol δ is presumed to function on the leading strand in the ΔN-Pol ε genetic background. However, the finding in this report that Pol α functions on the leading strand with CMG while Pol δ-PCNA is suppressed leaves open the possibility that Pol α could participate as the leading strand polymerase in ΔN-Pol ε mutants.

### Other aspects of replisome operations emanating from this study

Numerous proteins travel with the replication fork, and it is not possible to know a priori how many are needed for functional leading and lagging strand replication. This is the first study to reconstitute leading/lagging strand replication with three pure polymerases in a eukaryotic system. It reveals that many of the proteins that move with forks are not required to recapitulate leading/lagging strand synthesis in vitro. For example, Ctf4/AND-1 is essential in most cells (not in budding yeast), and yeast Ctf4 helps recruit Pol α bind to RPCs (Gambus et al., 2009), yet the current study shows that Pol α functionally interacts with CMG without Ctf4. Hence, the complete role of Ctf4 remains unknown. Likewise, the essential Mcm10 protein is known to bind Pol α (Warren et al., 2009), but this study shows that Mcm10 is not required for fork function in vitro. The same can be said for a myriad of proteins that form the RPC complex, including Mrc1, Csm3, Tof1, Topo I, Mcm10, Ctf4, and FACT complex (Gambus et al., 2006; Gambus et al., 2009). It is important to note, however, that in vitro fork progression is 4–5 ntds/s, 5–10 times slower than in vivo measurements, and thus one or more of these proteins may be required to increase the rate of fork movement.

The current report also reveals that Pol α can prime the leading strand directly. To our knowledge, before this report, all proposed models of leading strand initiation in bacteria and SV40 show priming on the lagging strand of a bidirectional origin, which becomes the leading strand of one fork, rather than directly priming the leading strands (Waga and Stillman, 1998; Méndez and Stillman, 2003; Kaguni, 2011). We show here that this is not necessary. Pol α is robust in initiating the leading strand directly and targets CMG to do so.

Cellular studies indicate that nucleosomes are involved in determining Okazaki fragment size (Smith and Whitehouse, 2012), but data in this report show that short Okazaki fragments of the size observed in vivo do not require nucleosomes. However, nucleosomes may still be needed for greater precision in Okazaki fragment length. We also demonstrate that Pol ε forms a stable and isolable complex with CMG, which stands in contrast to the inability to isolate Pol ε in RPCs as defined by a complex that stays associated during two successive affinity columns (Gambus et al., 2006). Presumably, the time involved in preparing RPC through two columns is sufficiently long for the CMGE complex to dissociate. However, some Pol ε can be recovered when isolated in a single-step pull down with epitope tagged CMG from cell extracts (Sengupta et al., 2013).

Reconstitution of cellular replisomes in vitro should provide a framework to explore the effects of other proteins that move with forks and of post-translational modifications that control eukaryotic forks in response to the cell cycle and DNA checkpoint mechanisms. Reconstituted systems should also enable detailed study of factors that maintain the epigenetic state of a cell during replication.

## Materials and methods

### Reagents and buffers

Radioactive nucleotides were from Perkin Elmer. Unlabeled nucleotides were from GE Healthcare. DNA modification enzymes and ϕ29 DNA polymerase were from New England Biolabs. DNA oligonucleotides were from Integrated DNA Technologies. Protein concentrations were determined using the Bio-Rad Bradford Protein stain and bovine serum albumin as a standard. Buffer A is 20 mM Tris-HCl, pH 7.5, 5 mM DTT, 0.1 mM EDTA, and 4% glycerol. Buffer B is the same as buffer A except 20 mM Tris-acetate, pH 7.5 was used in place of 20 nM Tris-HCl. Buffer C is 25 mM Tris-Cl pH 7.9, 10% glycerol, 1 mM DTT, 1 mM MgCl_2_, 5 mM imidazole, 20 mM KOAc, and 350 mM KCl. Buffer D is 25 mM Tris-OAc pH 7.6, 40 mM K-OAc, 40 mM K glutamate, 2 mM Mg-OAc_2_, 1 mM DTT, 20% glycerol, and 0.25 mM EDTA. Stop buffer is 1% SDS, 40 mm EDTA. Buffer H is 20 mM Hepes pH 7.5, 10% glycerol, 1 mM EDTA, 2 mM DTT, 350 mM KCl, 1 mM ATP, and 4 mM MgCl_2_.

### Proteins

Proteins were purified as described: RPA (Henricksen et al., 1994), *E. coli* SSB (Georgescu et al., 2011), PCNA (Yao et al., 2003), RFC containing full-length RFC1 (Finkelstein et al., 2003). Pol ε was expressed in yeast as described (Georgescu et al., 2014), in which a 3XFLAG tag was placed on the N-terminus of Pol2 in pRS425/GAL and transformed into yeast along with pJL6-expressing genes encoding Dpb2, Dpb3, and Dpb4 (Chilkova et al., 2003). All buffers were degassed before use to prevent oxidation of Fe-S centers. Pol δ was expressed and purified as described (Georgescu et al., 2014). CMG was purified as described (Georgescu et al., 2014). Ctf4 with a N-terminal 3XFLAG tag was expressed from a Gal1/10 promoter and purified from yeast using an anti-FLAG column, similar to methods described for Pol ε (Georgescu et al., 2014). Pol α was prepared by integrating the gene encoding Pol α with a C-terminal 3XFLAG tag into the yeast pRS402 vector under control of the Gal1/10 promoter, then integrated into strain OYO1 (*ade2-1 ura3-1 his3-11,15 trp1-1 leu2-3,112 can1-100 bar1Δ MAT*a, *pep4::KANMX6*), a strain constructed from W303 (a gift from Alan Tackett and Brian Chait, Rockefeller University) (Georgescu et al., 2014). The Pol12, Pri1, and Pri2 subunits were cloned into *E. coli* vectors pRSFDuet, pCDFDuet, and pACYCDuet/RIL, respectively (Novagen Inc., Madison, WI). Pol12 was transformed into *E. coli* BL21(DE3)codon plus RIL (Agilent, Santa Clara, CA), then induced with IPTG for 8 hr at 15°C. Pri1 and Pri2 were co-expressed in *E. coli* BL21(DE3) cells by IPTG induction for 8 hr at 15°C. A 12 l culture of induced yeast cells for Pol1 and 1 l of each induced *E. coli* cultures for Pol12 and Pri1 and Pri2 were co-crushed in a cryogenic mill as described for CMG (Georgescu et al., 2014). Frozen crushed cells were thawed at 12°C and the re-suspended with 20 ml of 250 mM Hepes pH 7.4, 1.25 M potassium glutamate, 5 mM EDTA. The lysate was spun at 42,000×*g* for 1 hr at 4°C. Anti-Flag agarose (1.2 ml) was added to the supernatant (80 ml) and incubated with slow rotation for 1.5 hr at 12°C. Beads were collected by centrifugation at 1500×*g*, washed twice with 5 ml 50 mM Hepes pH 7.4, 250 mM potassium glutamate, 1 mM EDTA, then loaded into a gravity column. The column was washed with 15 ml 50 mM Hepes pH 7.4, 250 mM potassium glutamate, 1 mM EDTA, 10% glycerol, and Pol α was eluted with 5 ml 50 mM Hepes pH 7.4, 250 mM potassium glutamate, 1 mM EDTA, 10% glycerol containing 20 μg/ml Flag peptide. The eluent was diluted to a conductivity equal to 150 mM NaCl using 25 mM Hepes pH 7.4, 1 mM EDTA, and 10% glycerol, then applied to a 1 ml Heparin agarose column. Pol α was eluted with a gradient of 100 mM to 1 M NaCl in 25 mM Hepes pH 7.4, 1 mM EDTA, 10% glycerol. Peak fractions were pooled, aliquoted, and stored at −80°C. Typical yield of Pol α was about 3 mg.

### Linear forked DNA substrate

To make the linear fork DNA substrate, a 3.2 kb sequence of DNA was designed such that one strand lacked dC residues and thus the other strand lacked dG. The G-rich strand was examined to eliminate runs of four G residues. The resulting 3260 bp sequence was synthesized by Biomatik (Wilmington, Delaware). The first four and last two bases represent overhangs generated by BsaI and BtsC1, respectively. Both of these enzymes cut outside of their recognition sequences, and their recognition sequences are excluded from the template.

CGGTATTCTAACCACATTAATCTACACCTCTCACACACTACTCATATCATCTTCCAAAACCCACCTTTAAAAAACCCTTTATCCACACTCATCACCATTTCACCAACCTTTTTCTTAATTCTACACAAATCCAATTAACCTATCTCCAATTTTAACTCCATCACCTCTTATATTAACCCACCTACTACTCCAACAATACCCTATCAAATCTACTTCTATCTCAAAACTATCACCTACTCCTTCCATCATAATCCACTCTTATCAATTAAACAATTATCCTTCTTTCCCACCATCACTCACCATCTTTTCTTAACACCCTTAACATTTCCTTTTATAAAACACTTCCAATCCTATTTTCTCACTATCCCACCCACCATAAAAACTATCTCACCCTAACTCAACCCTTTCCCTCTCACCAACACTCCTTTATCACACACACTTTACCACACAAATCCCTCCATCATACACCTTTACCCTCAAATCCTAAACCACCTAACTATTCCACACAATATATCTACAAAAATTTACTTTTCCACATCTCCAACCCTTCCAACACCTTAATCCCAAACCTTAACTAACCTCTCTTTAATACTTCCTCCCATTCCCACCACATACACCAAATTATCTTCAACTCAAAAACCTAAACTCTCCTTTTTATTCCCTATAAAAACTCTTAACCCTCCAATATACAACTCTAACTAACTTCATTATCAACCAATCTTCCTCTACTTCCTCATCTTATAATTTATCCATTCAAAATAACCTACCTACCACCTCTCCTCTTCACTTCCTACCCTAAAATCACCACCTTATCCCTAATTTACCTCTTTCAACTTTCCTTAACCCAACTTCTCAATCCTACTTCACTTACTTCTTATAAAACCATCATTATCACACTACACATTACTCTATCTATCCAATCATCACCTTCTACAATCCAAACTATCCCACTACCCTCTCATTCTACCTTTTCATCTATCTCAAACTATCCACCAATCCATACCTCAAACTTTAACCACCCACTCCAAATCTACAACATAAAATTAACCTATCACATTCCAAACTAATCACCCTAACCCTAACACCCTTTTATCCTCACCAAAATTACCATTTTCCTCTTTACTCATAAACAAACATTCTCACCCATTTATAAAACACTTAATACCCACTTAATTCACTTCCTTTTTCTACCTCACCATCATCAACTCCTAATATCAACAACCCAAAATCACCACCTATATCCTCATCTCCTATATAAAAAACTTCACATCTCAACCTCAAACCACCTATTCCACTTAATCCCAATCAACCTATCAACTCTACAACCTACTCTTCAAATACATCTCCTATCACTTTCCCACCTCCTTCAATCAATTATAACTTTATCACCTAAACATTCTAAAATCTCCTATCCACTACATCACATAAACCTAAACCTACTACCAACCTACCATTATCCTTATCAAACATCATCAATTCCATCTTTCTTTATACCCTCTCCATATCTCTCTCATTAAAAAAACCAAAATCTAACAACTTCTTATTTCTAATCAAAAAAACAATCAACCTAACTCATAAAATCTTCACCTTAAAATTCCTTTACATTTAACAAATCCACTCTCCCTATCTTTTTCATATCAAACTTATCTAAACCACTATCCTCATTTATCCTAATACTCCATATACAACACCAAATTTCTTATATCTCATCTAAAATCCTCCACCAATATAAACTCCTCTTTACCATTTCCACTCAACACACCAAATCTTATTATTCCATCAAATCTAATCCATTACCATCATCAACCCTAATAAACCTACTTCCCAACTTTTATCTCTCCACTACCACACCAAAAATTAACCTCCTCTAAAAACTATCATTCCCTTTACCTCTTCCACATTCCACCTATAACTCCTCATCTTAAAACCAATCAACACCAAACAACTATCTCACCATATTTCCTCTCCAAACCAACAAATTAACAATCCTACCCACTCCAACCTCCACATTACTAATAACTAAACTTACCTTACCTACCACACCCTATCAACCATATTTAAAAAATTACTTATTCACTAACTAAAACATCACCCACAAACTTAAATCATCACCTCCTCTTTTCCACCTTATTCACCAACCCAATCTATCTATCTCACCTATACCTTTCCCTAATATCTTTTACTAACCCTATAAATACCACAATTCTAAAAAACCCATACTTATCTCACACATCACTTTATACTTCACTCTTAAAATACCCTCCAATATATATTACAACCCAAAAATATCTCCCTTCTATCTCCTACACACAAATTATACCACTTTTAAACCACTCCTCATCTCTAACCCAACCCTCTACAATTCCATACATCTCTACTATCAACATCACTCCTTCTTTTCCACTCTTCTCTCCACATCTTTATTAAACATCTCCTCCTCATTTTCACATAACTATTTACTAAATAAATTTACCTAAACTACATTTATTAAAACCCTACAACATACTCCTTATTCTCCTACCTACCATTCTCTAATCTCTTTACATTCTACTACTTTCCTACCTACTATCTCAATAAATTCACTTTCCTTTCACCACACACCAACACACCTTCCTCCAAATTCTTTATATCTCCTTCTCCTAACCAAATTCCTCACTAATAACATCTTACCTCCCTACCTTTTTCCTTCTACCCTCCACCATTTCCCAACCTCATACTCAATAATCAATTTACCCTCCCACAACATAACTCTATTAACACCCATTTTCTATCCATCAACTTCCTATTACTTAAATTATCTTTTAAACCAATAAAACTCCACCTCAACCACCCACCTCTCTCTTTCAATCCAATTTCAATCTTTCCAACCATTCTATCTACCCTAAACTATTAACTATCTATCTCACCCACAATCCCTCCTACAATTCACAACAACATTCCACCACTTCACTTTATCTTCACCTCCAAACTTATTCCTTCCCATTATCACCTTCTCCAAAACCCACAAACATCTAACTCTCATCTCTCAACACTTTCTACCCATTCTCTCTAACAAAATTCCCTTACTCTTTATTCACAAAACCACTAAAATCACCACAACAACCCAACAAAAACAAAATCCTCACTTACCTATACTCAATAAATCCTTCAACTCATTATTCTATTTCTAACCCTAAATCAAAACTCCCATATCTACCATTCTTTCCACTCAATTAAATCCCACCAACCCTTATTTTCCTCCAATAACTTAACC.

The synthetic 3.2 kb DNA was cut from the plasmid using BsaI-HF and BtsCI and purified from low-melting agarose gel. 35 pmol of the 3.2 kb linear DNA template was ligated to a fivefold excess of forked junction on the BsaI end and to a 10-fold molar excess of a short blocking duplex on the BtsCI end. To make the forked junction, 1050 pmol 1T oligonucleotide was annealed to 175 pmol 160mer oligonucleotide, as described (Georgescu et al., 2014). The 160mer is the leading strand template and contains a 3′ biotin and three 3′ terminal thiophosphate linkages, to protect against nucleases. The synthetic fork contains a 5′-phosphorylated-ACCG overhang in the duplex region that ligates to the BsaI end of the 3.2 kb nucleotide-biased duplex. To protect the BtsCI end of the 3.2 kb duplex from excision and fill-in by the proofreading exonucleases of Pols δ and ε, a short protecting duplex was ligated to the BtsCI end of the forked junction. The blocking duplex was formed upon annealing 350 pmol 5′-tggttagtatagcaagtagagg-3′ and 2100 pmol tctacttgctatactaaccat3′-′3-dT. After ligation, this results in a 3.2 kb duplex with two 5′ terminal nucleotides at one end, to resist digestion by 3′–5′ exonuclease inherent in the DNA polymerases. Both the blocking duplex and the synthetic forked junction were present in the ligation reaction. Ligation was for 18 hr at 15°C with T4 DNA ligase. Excess non-ligated oligonucleotides were removed by gel filtration over a 20 ml bed volume of Sepharose 4B (GE Healthcare, Piscataway, NJ) equilibrated in 10 mM Tris-acetate pH 7.5, 1 mM EDTA, and 100 mM sodium acetate, pH 7.44. Peak fractions containing the nucleotide-biased linear forked DNA were pooled and stored at −20°C. When the substrate was primed, a DNA oligonucleotide was annealed to the leading strand template, DNA 37mer (C2) as described (Georgescu et al., 2014).

### Leading/lagging strand replication assays

Replication assays contained 1.5 nM linear forked DNA, 24 nM CMG, 400 nM RPA (unless noted otherwise), 20 nM PCNA (unless noted otherwise), 6 nM RFC (unless noted otherwise), and Pol α, Pol ε, and Pol δ as indicated in the figure legends, in 25 mM Tris-acetate pH 7.5, 10 mM Mg-acetate, 50 mM potassium glutamate, 5 mM DTT, 0.1 mM EDTA, 40 μg/ml BSA, 0.1 mM AMP–PNP, 5 mM ATP, 200 μM each rCTP, rUTP, rGTP, 60 μM of each unlabeled dNTP, and 20 μM of the labeled dNTP. Reactions were staged as follows. CMG was added first and pre-incubated with the DNA and 0.1 mM AMP-PNP for 10 min at 30°C, then the noted polymerases together with the RFC and PCNA (where indicated) were added, along with dATP, dCTP for an additional 2 min. Replication was then initiated upon the addition of a solution containing the RPA, ATP, dTTP, and dGTP. It is important to note that for leading strand replication reactions, we used 20 μΜ dCTP and 10 μCi ^32^P-dCTP, while for lagging strand replications, we used 20 μM dGTP and 10 μCi ^32^P-dGTP. Exceptions to this protocol are noted in the figure legends. Timed aliquots were removed and quenched upon adding SDS and EDTA to final concentrations of 0.5% and 20 mM, respectively. Quenched reactions were analyzed in 0.7% or 2% alkaline agarose gels and imaged in a Typhoon 9400 PhosphorImager (GE/Molecular Dynamics, Berkeley, CA).

In order to compare total DNA synthesis rates on leading and lagging strand, we performed standard replication reactions containing all three DNA polymerases at 10 nM, CMG (30 nM), RFC (10 nM), PCNA (20 nM), and RPA (400 nM) in presence of 1 mM ATP, rNTPs (C,G,U at 100 μM), and 30 μM dNTPs; reactions were divided, and either ^32^P-dCTP (leading) or ^32^P-dGTP (lagging) was added. Timed reactions were stopped with an equal volume of 2× STOP solution (40 mM EDTA and 1% SDS) and spotted on DE81 filter papers, then analyzed using a Perkin Elmer Liquid Scintillation Analyzer (Perkin Elmer, Tri-Carb 2910 TR). Separately, we performed control replication reactions using ϕX174 ssDNA coated with RPA, containing a known amount of Gs and Cs, confirm that ^32^P-dCTP and ^32^P-dGTP are equally incorporated by each of the DNA polymerases in our experimental conditions.

### Restriction enzyme analysis of Okazaki fragment distribution

Three replication reactions were performed as described above with the following differences. The first reaction used 1.5 nM unprimed forked DNA and 10 nM Pol α with 20 μM dGTP as well as 10 μCi ^32^P-dGTP to label the lagging strand; the second reaction utilized 1.5 nM unprimed forked DNA and 10 nM each Pol ε and Pol α along with 20 μM dGTP and 10 μCi ^32^P-dGTP to label the lagging strand, and the third reaction contained DNA-primed forked DNA and 1 U of ϕ29 DNA polymerase along with 20 μM dCTP and 10 μCi ^32^P-dCTP to label the leading strand. Each reaction was quenched upon heating to 65°C for 10 min to inactivate the CMG and polymerases. Then, reactions were divided into three tubes, one was untreated, the second was treated with EarI, and the third was treated with PsiI, adjusting the reaction buffer for each enzyme with the commercial provided buffer. Reactions were analyzed in a 2% native agarose gel followed by autoradiography in a Typhoon 9400 PhosphorImager (GE/Molecular Dynamics).

### Primed ssDNA reactions

Reactions contained 1.5 nM ϕX174 circular ssDNA (as circles) primed with a DNA 30-mer and pre-incubated for 10 min with 420 nM RPA (as heterotrimer) in 20 mM Tris-Cl (pH 7.5), 50 mM potassium glutamate, 5 mM DTT, 0.1 mM EDTA, 40 μg/ml BSA, 8 mM MgOAc, 0.5 mm ATP, 5% glycerol, and 60 μM, each of dGTP and dATP. Reactions also contained the indicated amounts of RFC, PCNA, and Pol α and were pre-incubated for 5 min at 30°C. DNA synthesis was initiated by adding 15 μl of 60 μM dCTP, 20 μM dTTP, 15 μCi of (α-^32^P) dTTP and incubated at 30°C. At the times indicated, 25-μl aliquots were removed and quenched by addition of an equal volume of 1% SDS/40 mm EDTA. Products were analyzed in 0.7% alkaline agarose gels. Gels were dried, exposed to PhosphorImager screens, and imaged using a Typhoon 9400 PhosphorImager (GE/Molecular Dynamics).

### Bead-based protein complex analysis

Proteins were premixed in the following amounts: 80 pmol StrepTag-Pol ε, 120 pmol CMG, and when present, 200 pmol Ctf4 (as trimer). Proteins were brought to a final volume of 200 μl in 100 mM sodium phosphate, 150 mM NaCl pH 8.0, and incubated with 50 μl (as a 10% slurry) Strep-Tactin magnetic beads (Qiagen, Valencia, CA) for 1 hr at 4°C with end-over-end mixing. Beads were collected in a magnetic separator and washed twice in 200 μl 100 mM sodium phosphate, 150 mM NaCl pH 8.0, then eluted in 75 μl 10 mM biotin in 100 mM sodium phosphate, 150 mM NaCl pH 8.0 for 20 min on ice. Samples were analyzed in 8% SDS-PAGE followed by staining with Coomassie Blue Denville stain.
